# Nal-IRI with 5-fluorouracil (5-FU) and leucovorin or gemcitabine plus cisplatin in advanced biliary tract cancer - the NIFE trial (AIO-YMO HEP-0315) an open label, non-comparative, randomized, multicenter phase II study

**DOI:** 10.1186/s12885-019-6142-y

**Published:** 2019-10-23

**Authors:** L. Perkhofer, A. W. Berger, A. K. Beutel, E. Gallmeier, S. Angermeier, L. Fischer von Weikersthal, T. O. Goetze, R. Muche, T. Seufferlein, T. J. Ettrich

**Affiliations:** 1 Department of Internal Medicine I, Ulm University, Albert-Einstein-Allee 23, 89081 Ulm, Germany; 2 Department of Internal Medicine and Gastroenterology, Klinikum im Friedrichshain, Landsberger Allee 49, 10249 Berlin, Germany; 3 Department of Gastroenterology and Endocrinology, University of Marburg, Baldingerstraße, 35043 Marburg, Germany; 4 Internal Medicine I, Klinikum Ludwigsburg, Posilipostraße 4, 71640 Ludwigsburg, Germany; 5 Gesundheitszentrum St. Marien, Mariahilfbergweg 7, 92224 Amberg, Germany; 6 Institute of Clinical Cancer Research (IKF) at Krankenhaus Nordwest, UCT-University Cancer Center, Steinbacher Hohl 2-26, 60488 Frankfurt, Germany; 7 Institute of Epidemiology and Medical Biometry, Ulm University, Schwabstraße 13, 89081 Ulm, Germany

**Keywords:** Biliary tract cancer, Cholangiocarcinoma, Chemotherapy, Nanoliposomal-irinotecan, Palliative treatment

## Abstract

**Background:**

Biliary tract cancer (BTC) has a high mortality. Primary diagnosis is frequently delayed due to mostly unspecific symptoms, resulting in a high number of advanced cases at the time of diagnosis. Advanced BTCs are in principle chemotherapy sensitive as determined by improved disease control, survival and quality of life (QoL). However, median OS does not exceed 11.7 months with the current standard of care gemcitabine plus cisplatin. Thereby, novel drug formulations like nanoliposomal-irinotecan (nal-IRI) in combination with 5- fluorouracil (5-FU)/leucovorin may have the potential to improve therapeutic outcomes in this disease.

**Methods:**

NIFE is an interventional, prospective, randomized, controlled, open label, two-sided phase II study. Within the study, 2 × 46 patients with locally advanced, non-resectable or metastatic BTC are to be enrolled by two stage design of Simon. Data analysis will be done unconnected for both arms. Patients are allocated in two arms: Arm A (experimental intervention) nal-IRI mg/m^2^, 46 h infusion)/5-FU (2400 mg/m^2^, 46 h infusion)/leucovorin (400 mg/m^2^, 0.5 h infusion) d1 on 14 day-cycles; Arm B (standard of care) cisplatin (25 mg/m^2^, 1 h infusion)/gemcitabine (1000 mg/m^2^, 0.5 h infusion) d1 and d8 on 21 day-cycles. The randomization (1:1) is stratified for tumor site (intrahepatic vs. extrahepatic biliary tract), disease stage (advanced vs. metastatic), age (≤70 vs. > 70 years), sex (male vs. female) and WHO performance score (ECOG 0 vs. ECOG 1). Primary endpoint of the study is the progression free survival (PFS) rate at 4 months after randomization by an intention-to-treat analysis in each of the groups. Secondary endpoints are the overall PFS rate, the 3-year overall survival rate, the disease control rate after 2 months, safety and patient related outcome with quality of life. The initial assessment of tumor resectability for locally advanced BTCs is planned to be reviewed retrospectively by a central surgical board. Exploratory objectives aim at establishing novel biomarkers and molecular signatures to predict response. The study was initiated January 2018 in Germany.

**Discussion:**

The NIFE trial evaluates the potential of a nanoliposomal-irinotecan/5-FU/leucovorin combination in the first line therapy of advanced BTCs and additionally offers a unique chance for translational research.

**Trial registration:**

Clinicaltrials.gov NCT03044587. Registration Date February 7th 2017.

## Background

Biliary tract cancer (BTC) is a rare type of cancer and ranks beyond 10th in Western World tumor incidence [1]. However, the incidence particularly of intrahepatic BTC is rising, [2, 3] resulting BTC to be the 5th leading cause of cancer related deaths [1]. The main reason for the high mortality of BTCs can be found in the generally advanced stage at primary diagnosis, due to often missing early symptoms [4]. 5-year overall survival rates do not exceed 5% for patients with advanced or metastatic disease [1]. Advanced BTCs respond to chemotherapy, resulting in an improved disease control rate, survival time and quality of life (QoL) [5–7]. However, overall survival rates beyond 10 months remain rare in the palliative setting. The current standard of care combines conventional chemotherapeutic agents for patients who are in a good performance status. Therapy is based on the ABC-02 phase III trial that demonstrated a beneficial progression-free (PFS) and overall survival (OS) for a combination of gemcitabine plus cisplatin compared to gemcitabine alone (Cis + Gem vs. Gem: OS 11.7 vs. 8.1 months; PFS 8.0 vs. 5.0 months) [6]. However, the therapeutic landscape in oncology is steadily evolving bringing novel compounds into daily clinical routine in various cancer entities. Several antibodies and inhibitors like cetuximab or sorafenib were evaluated in advanced BTC, but failed to improve outcome [5, 8]. Irinotecan combined with 5-FU showed promising results in the 1st- [9] and 2nd-line treatment [10] of advanced BTC and is commonly used as therapeutic option after failure of the 1st-line therapy with gemcitabine/cisplatin. Consequently, encapsulation of irinotecan in pegylated liposomes could be of value in advanced BTC as efficacy and tolerability of this drug are already proven in a number of solid tumors including pancreatic [11], gastric [12] and colorectal cancers [13]. Nanoliposomal-irinotecan (nal-IRI) showed extended plasma half-life and increased intratumoral concentrations compared to conventional irinotecan in preclinical models [14–16]. The NAPOLI-1 trial transferred this to the patient and demonstrated in a phase III setting a significantly prolonged OS for 2nd-line therapy with nal-IRI/5-fluorouracil (5-FU)/leucovorin (LV) in patients with metastatic pancreatic cancer compared to 5-FU/LV only [11]. The superiority shown in the NAPOLI-1 trial provides compelling evidence for a potential efficacy in advanced BTC. The toxicity profile of nal-IRI is similar to what has been described for irinotecan that is routinely used in clinical practice by oncologists [12].

The NIFE phase II trial aims to challenge the current palliative first-line therapy of BTC by use of nanoliposomal-irinotecan/5-FU/leucovorin and to further establish specific biomarker signatures.

## Methods and study design

NIFE is an interventional, prospective, randomized, controlled, open label, two-sided phase II study, using the optimal two-stage design of Simon in each of the experimental arms.

### Study objectives

#### Primary objective

- PFS rate at 4 months, defined as the proportion of patients with non-progressive disease 4 months after randomization by intention-to-treat analysis

*Secondary objectives:*

- Overall progression-free survival
- 3-years overall survival
- Disease control rate according to RECIST 1.1 [17] after 2 months
- Objective tumor response rate (ORR) according to RECIST 1.1 [17]
- Toxicity/safety according to CTCAE-criteria version 4.03 (≥ Grade 3/4)
- Patient-related outcome/quality of life/time to definitive deterioration (TUDD) to be assessed with the following tools: EORTC QLQ-BIL21, QLQ-C30 and HADS-D
- Tumor resectability in accordance with a retrospective central surgical board compared to local surgical review
- Radiological response according to RECIST 1.1 [17] and volumetry determined by a retrospective central radiological review

*Exploratory objectives:*

- Exploratory biomarkers analysis (cfDNA exome sequencing, transcriptome, miRNA-arrays prior to and after start of treatment, and on progression).
- Establishment of predictive/prognostic biomarker profiles for advanced BTC
- Tumor evolution under chemotherapy

#### Patient selection and randomization

**Fig. 1:**
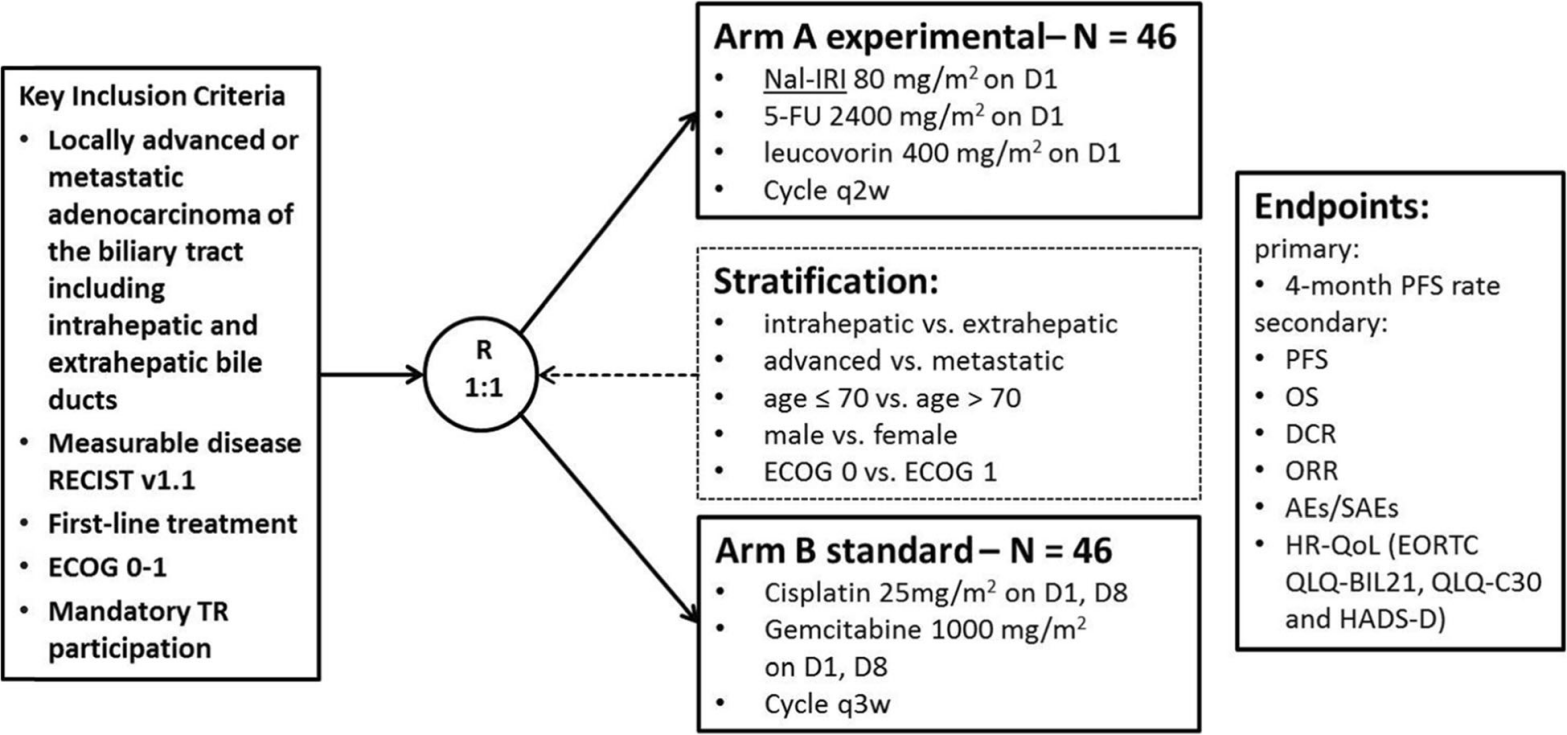
Flow diagram NIFE trial

Approximately 120 patients have to be screened to get 92 randomized patients (46 patients per arm). In the study, 30 participating centers are planned. The trial is randomized in a 1:1 ratio to the experimental (Arm A) or standard arm (Arm B) to get comparable sample sizes by stratified permutated block randomization to avoid a selection bias, see Fig. 1. The randomization (1:1) is stratified for tumor site (intrahepatic vs. extrahepatic biliary tract), disease stage (advanced vs. metastatic), age (≤70 vs. > 70 years) [18], sex (male vs. female) and WHO performance score (ECOG 0 vs. ECOG 1).

#### Main inclusion and exclusion criteria

*Inclusion criteria:*

- Histologically confirmed, non-resectable, locally advanced or metastatic adenocarcinoma of the intrahepatic or extrahepatic biliary tract (not papillary cancer or gallbladder cancer)
- Non-resectability has to be stated by the local multidisciplinary tumor board
- Measurable or assessable disease according to RECIST 1.1 [17]
- ECOG performance status 0–1
- Age ≥ 18 years at time of study entry
- Life expectancy of more than 3 months
- If applicable, adequately treated biliary tract obstruction before study entry with total bilirubin concentration ≤ 2 x ULN
- Adequate blood count, liver-enzymes, and renal function:
  - ◦ AST (SGOT)/ALT (SGPT) ≤ 5 x institutional upper limit of normal
  - ◦ Serum Creatinine ≤1.5 x institutional ULN and a calculated glomerular filtration rate ≥ 30 mL per minute
  - ◦ Patients not receiving therapeutic anticoagulation must have an INR < 1.5 ULN and PTT < 1.5 ULN within 7 days prior to randomization
- No prior palliative chemotherapy for biliary tract cancer
- No adjuvant treatment within 6 months prior to study entry
- Written informed consent including participation in translational research

*Exclusion criteria:*

- Clinically significant cardiovascular disease (incl. Myocardial infarction, unstable angina, symptomatic congestive heart failure, serious uncontrolled cardiac arrhythmia) within 6 months before enrollment
- Prior (< 3 years) or concurrent malignancy (other than biliary-tract cancer) which either progresses or requires active treatment. Exceptions are: basal cell cancer of the skin, pre-invasive cancer of the cervix, T1a or T1b prostate carcinoma, or superficial urinary bladder tumor [Ta, Tis and T1].
- Known Gilbert-Meulengracht syndrome
- Known chronic hypoacusis, tinnitus or vertigo
- Previous enrollment or randomization in the present study (does not include screening failure).

#### Staging assessments

- Medical history and demographics including dates and description of initial diagnosis of advanced biliary tract cancer and relevant concurrent illness
- Complete physical examination including: weight, height, BSA, vital signs (blood pressure, heart rate, respiratory rate and oral body temperature)
- Residual symptoms/toxicities from previous therapies should be recorded according to the NCI Common Toxicity Criteria
- ECOG Performance Status
- Review of prior/concomitant medications
- Tumor assessment according to RECIST 1.1 [17] done by local investigator in the context of standard care (contrast enhanced multislice CT of the abdomen or abdominal MRI and an enhanced multislice thoracic CT scan)
- EORTC QLQ-BIL21, QLQ-C30 and HADS-D questionnaire
- Nutritional risk score
- 12-lead ECG
- Hematological tests, Clinical chemistry
- Serum Tumor Marker (Ca 19–9, CEA)

#### Treatment

Treatment is planned in an outpatient setting for all study drugs and will continue until there is evidence of disease progression or occurrence of any other discontinuation criterion. If nal-IRI or cisplatin have to be discontinued permanently under therapy for a reason other than progressive disease, treatment should continue with the remaining drug in the trial, with full adherence to all protocol-related requirements. Within a therapy cycle, treatment should continue on schedule, but a variance of ±5 days may be allowed to accommodate holidays, weekends or other justifiable events.

*Arm A (experimental arm):*

- Nanoliposomal-irinotecan 80 mg/m^2^ as 1.5 h infusion
- 5-fluorouracil 2400 mg/m^2^ as 46 h infusion
- Leucovorin 400 mg/m^2^ as 0.5 h infusion
- Cycle q2w ± 5 days

*Arm B (standard arm):*

- Cisplatin 25 mg/m^2^ as 1 h infusion on day 1 and day 8
- Gemcitabine 1000 mg/m^2^ as 0.5 h infusion on day 1 and day 8
- Cycle q3w ± 5 days

#### Follow-up

All subjects undergo follow-up for survival until the end of the study irrespective of subsequent treatments, or until the sponsor ends the study (follow-up extension phase). Patient contact is to be established by telephone interview or face-to-face, whichever prevails.

The following procedures will be performed during follow-up every 8 weeks:

- Assessment of survival status
- Anti-cancer treatments must be recorded during follow up
- Reporting of all adverse events (AEs) and severe adverse events (SAEs) within 4 weeks after the end of treatment (EoT) visit

#### Sample size calculation and statistical analysis

Simon’s optimal two-stage design was used for sample size calculation for each group by OneArmPhaseTwoStudy software [19]. H_0_: less than 40% of patients are progression-free by 4 months of nal-IRI plus 5-FU/leucovorin. Alternative hypothesis: ≥60% of patients are progression-free by 4 months of nal-IRI plus 5-FU/leucovorin. If 7 or less of the first 18 patients assigned to nal-IRI plus 5-FU/leucovorin have a tumor response or stable disease at 4 months, H_0_ will be accepted and the study will be terminated. If 8 or more patients with tumor response or stable disease are observed, another 28 patients in each treatment group are to be included. At the final analysis, H_0_ will be accepted if less than 23 of the total 46 patients in the nal-IRI plus 5-FU/leucovorin group had a tumor response or stable disease at 4 months. With this design, alpha = 10% (significance level) and power = 90%. As the study will be analyzed as intention-to-treat analysis (ITT), all patients will be analyzed (missing data will be considered as failure). Hence, a sample size of *n* = 46 per treatment arm and a total *N* = 92 enrolled and randomized patients is required. It is assumed that approx. 120 patients need to be screened for eligibility.

#### Quality of life assessment and time to definitive deterioration

Health related quality of life (HRQL) will be assessed by using the EORTC QLQ-C30 questionnaire version 3.0. The questionnaire contains 5 functions (physical, role, cognitive, emotional, and social), 9 symptoms (fatigue, pain, nausea and vomiting, dyspnea, loss of appetite, insomnia, constipation, diarrhea and financial difficulties) and the global health status/quality of life (GBH/QoL) [20]. To further specify the assessment the module for biliary tract cancer (QLQ-BIL21) with 21 items related to disease symptoms, treatment side effects and emotional issues in BTC is included [21]. A calculation of the median time to definitive deterioration (TUDD) using the EORTC QLQ-C30 questionnaire data is planned. The TUDD will be calculated in accordance to Anota et al. and Bonnetain et al. and is defined as an ongoing deterioration of at least 5 points compared to the baseline [22, 23]. The emotional and social impact of being diagnosed with BTC is highly relevant. To detect anxiety and depression, which are the most common co-morbidities of physical illness, the HADS-D questionnaire (Hospital Anxiety and Depression Scale – German version) is used. The HADS-D has 14 items (7 anxiety, 7 depression) each with a 4-point verbal rating scale scored from 0 to 3. The scale deliberately avoids physical indicators of mental disorders (e.g., insomnia, weight loss) and severe psychopathological symptoms allowing high sensitivity with proven psychometric quality criteria [24, 25].

HRQL should be assessed at following time points:

- At baseline, within 7 days prior to randomization
- Before the beginning of each cycle of systemic therapy
- At end of treatment visit
- Quality of life assessment should be performed even when chemotherapy cannot be given at the beginning of a cycle e.g. due to toxicity reasons.

#### Nutritional screening

The nutritional risk score (NRS) questionnaire will be used for the evaluation of nutritional anomalies. Malnutrition and weight loss are common problems in advanced BTC patients and contribute to morbidity and mortality. Furthermore, tolerance of chemotherapy is often worse in patients with severe malnutrition. The NRS questionnaire is a simple tool to screen patients for malnutrition [26]. The questionnaires will be completed at time of screening, every 8 weeks under therapy and at the EoT visit.

#### Translational research

This trial provides the opportunity to systematically obtain biologic material from therapy naive patients suffering from advanced BTC for comprehensive molecular characterization. It allows to assess treatment associated tumor evolution under 1st-line palliative chemotherapy with different regimens. Consequently, we will collect tissue samples obtained for initial diagnosis for exome sequencing best versus worst responders. We hypothesize that exome sequencing of microdissected tumor cells from initially taken core biopsies will identify important biologic differences between tumors responding to cytotoxic chemotherapy compared to those not responding to the treatment and thereby provide potential predictive markers. In parallel, blood samples of each patient will be taken prior to treatment, after 4–5 weeks of treatment, thereafter in parallel to radiologic tumor assessments until disease progression (radiologically confirmed). Circulating cell-free tumor DNA will be extracted and analyzed by targeted genotyping in order to verify the potential of liquid biopsy as a disease diagnosis and treatment monitoring tool, as previously shown. Mutation profiles obtained from tissue and blood will be compared to evaluate whether tumor DNA analysis from blood yields a pattern comparable to tumor tissue and could be used to establish “easy to obtain” prognostic and predictive markers for nal-IRI based treatment.

#### Ethical aspects, trial registration

All patients have to sign written informed consent including participation in translational research and any locally-required authorization (including EU Data Privacy Directive in the EU, Declaration of Helsinki) obtained from the subject prior to performing any protocol-related procedures, including screening evaluations. The ethics committee of Ulm University approved the NIFE-trial as leading ethics committee for all German sites according to German regulative laws for trials (Arzneimittelgesetz). In addition, local ethics committees approved the participating sites. The trial is registered with ClinicalTrials.gov (NCT0344587).

## Discussion

Median overall survival in patients with advanced BTC is still devastating, generally not exceeding 1 year with the current therapeutic concepts. The results of the ABC-02 ^6^ and the BINGO trial [5] defined gemcitabine/cisplatin (or oxaliplatin) as treatment of choice in advanced BTC first line therapy. Therefore the investigators reported a progression-free survival (PFS) rate of 54% at 4-months in the gemcitabine/oxaliplatin group. Irinotecan was evaluated in several combinations in advanced BTC as first-line treatment, [27–30] showing the most promising results in combination with a thymidylate synthase inhibitor [31–33]. There is evidence that the nanoliposomal formulation of irinotecan may confer improved efficacy of the drug [14, 15, 34–37]. This encouraged us to try nal-IRI/5-FU/leucovorin in the first line treatment of advanced BTC, particularly given the positive data on safety and tolerability in both phase II and III trials as well as in real-life data in PDAC [11, 38, 39]. The NIFE trial aims to update and widen the treatment landscape in advanced BTC by using Nal-IRI/5-FU/leucovorin. For the NIFE trial we assume that ≥60% of patients are progression-free after 4 months of nal-IRI/5-FU/leucovorin. An interim analysis is planned after 18 patients have been enrolled to confirm the hypothesis.

The knowledge on BTC biology is still limited compared to other solid cancers. Recent sequencing studies shed more light on the mutational landscape of BTC and encourage the use of novel therapeutic targets [40–42]. However, a synergistic chemotherapy backbone is commonly needed like in other difficult to treat GI malignancies [43, 44]. Thereby, advanced BTC already showed the limitations of such strategies with no effects by adding cetuximab to standard chemotherapy in the BINGO trial [5]. Anyhow the spectrum of BTC mutations appears to lie within other gastrointestinal epithelial cancers with similar oncogenic mutations [42, 45, 46]. As a consequence a proper definition of BTC subtypes is paramount potentially guiding future treatment approaches. Therefore an expanded liquid biopsy program like that included in the NIFE trial may allow new insights on stratification and especially on the development of the mutational landscape under therapy.

To sum up, the NIFE trial evaluates the potential of nanoliposomal-irinotecan/5-FU/leucovorin in the first line therapy of advanced BTCs and additionally offers a unique chance for translational research.

## Data Availability

Not applicable. Data sharing is planned once the trial is completed.

## Abbreviations

(m)DFS: (median) disease free survival
(m)OS: (median) overall survival
(m)PFS: (median) progression free survival
5-FU: 5- fluorouracil
AE: Adverse event
AIO: Arbeitsgemeinschaft Internistische Onkologie
BSA: Body surface area
BTC: Biliary tract cancer
CBC: Complete blood count
cfDNA: Circulating free DNA
Cis: Cisplatin
CT: Computed Tomography
CTC: Common toxicity criteria
CTCAE: Common Terminology Criteria for Adverse Events
DCR: Disease control rate
ECG: Electrocardiogram
ECOG: Eastern Cooperative Oncology Group
EORTC: European Organization for Research and Treatment of Cancer
EOT: End of treatmend
FOLFIRINOX: Fluorouracil leucovorine, irinotecan, oxaliplatin
GBH: Global health status
G-CSF: Granulocyte-colony stimulating factor
Gem: Gemcitabine
HADS-D: Hospital Anxiety and Depression Scale
HR: Hazard ratio
HRQL: Health related quality of life
ITT: Intention to treat
LV: Leucovorin
MRI: Magnetic Resonance Imaging
Nab-Paclitaxel: Nano albumin bound Paclitaxel
Nal-IRI: Nanoliposomal-Irinotecan
NCI: National Cancer Institute
NRS: Nutritional risk score
ORR: Objective response rate
PDAC: Pancreatic ductal adenocarcinoma
QLQ-C30: Quality of life questionnaire-core 30
QoL: Quality of life
RECIST: Response Evaluation Criteria in Solid Tumors
SAE: Severe adverse event
TUDD: Time until definitive deterioration
ULN: Upper limit of normal
WHO: World health organization
